# Selective Vulnerability of the Nucleus Basalis of Meynert Among Neuropathologic Subtypes of Alzheimer Disease

**DOI:** 10.1001/jamaneurol.2019.3606

**Published:** 2019-10-28

**Authors:** Fadi S. Hanna Al-Shaikh, Ranjan Duara, Julia E. Crook, Elizabeth R. Lesser, Jolien Schaeverbeke, Kelly M. Hinkle, Owen A. Ross, Nilufer Ertekin-Taner, Otto Pedraza, Dennis W. Dickson, Neill R. Graff-Radford, Melissa E. Murray

**Affiliations:** 1 Department of Neuroscience, Mayo Clinic, Jacksonville, Florida; 2 Wien Center for Alzheimer’s Disease and Memory Disorders, Mount Sinai Medical Center, Miami Beach, Florida; 3 Department of Health Sciences Research, Mayo Clinic, Jacksonville, Florida; 4 Department of Neurology, Mayo Clinic, Jacksonville, Florida; 5 Department of Psychiatry and Psychology, Mayo Clinic, Jacksonville, Florida

## Abstract

**Importance:**

Corticolimbic patterns of neurofibrillary tangle (NFT) accumulation define neuropathologic subtypes of Alzheimer disease (AD), which underlie the clinical heterogeneity observed antemortem. The cholinergic system, which is the target of acetylcholinesterase inhibitor therapy, is selectively vulnerable in AD.

**Objective:**

To investigate the major source of cholinergic innervation, the nucleus basalis of Meynert (nbM), in order to determine whether there is differential involvement of NFT accumulation or neuronal loss among AD subtypes.

**Design, Setting, and Participants:**

In this cross-sectional study, retrospective abstraction of clinical records and quantitative assessment of NFTs and neuron counts in the nbM was completed in January 2019 at the Mayo Clinic using the Florida Autopsied Multi-Ethnic (FLAME) cohort, which had been accessioned from 1991 until 2015. The FLAME cohort is derived from the deeded autopsy program funded throughout the State of Florida’s memory disorder clinic referral services. Of the 2809 consecutively accessioned FLAME cohort, 1464 were identified as neuropathologically diagnosed AD cases and nondemented normal controls available for clinicopathologic assessment. Quantification of NFTs and neuronal density in the anterior nbM was performed blinded to neuropathologic groupings.

**Main Outcomes and Measures:**

Demographic and clinical characteristics, including cognitive decline measured using the Mini-Mental State Examination score (range, 0-30), were evaluated. The anterior nbM was investigated quantitatively for neuronal loss and NFT accumulation.

**Results:**

In total, 1361 AD subtypes and 103 nondemented controls were assessed. The median (interquartile range) age at death was 72 (66-80) years in hippocampal sparing (HpSp) AD, 81 (76-86) years in typical AD, and 86 (82-90) years in limbic predominant AD. The median (interquartile range) count per 0.125 mm^2^ of thioflavin S–positive NFTs was highest in the nbM of HpSp AD (14 [9-20]; n = 163), lower in typical AD (10 [5-16]; n = 937), and lowest in limbic predominant AD (8 [5-11], n = 163) (*P* < .001). The median (interquartile range) neuronal density per millimeters squared was lowest in HpSp AD cases (22 [17-28]; n = 148), higher in typical AD (25 [19-30]; n = 727), and highest in limbic predominant AD (26 [19-32]; n = 127) (*P* = .002). Multivariable regression modeling of clinical and demographic variables was performed to assess overlap in NFT accumulation and neuronal density differences among AD subtypes. Higher NFT accumulation in the nbM was associated with younger age at onset for HpSp AD (β, −1.5; 95% CI, −2.9 to −0.15; *P* = .03) and typical AD (β, −3.2; 95% CI, −3.9 to −2.4; *P* < .001). In addition, higher NFT accumulation in the nbM of typical AD cases was associated with female sex (β, 2.5; 95% CI, 1.4-3.5; *P* < .001), apolipoprotein E ε4 allele (β, 1.3; 95% CI, 0.15-2.5; *P* = .03), and lower Mini-Mental State Examination scores (β, −1.8; 95% CI, −3.2 to −0.31; *P* = .02). Demographic and clinical progression variables were not associated with NFT accumulation in the nbM of limbic predominant AD cases.

**Conclusions and Relevance:**

These data provide supportive evidence that NFT accumulation in the nbM may underlie more widespread and severe cholinergic deficits in young-onset AD, in particular in patients with HpSp AD. Moreover, these findings underscore the importance of considering age at onset, sex, and apolipoprotein E genotype when assessing outcomes in AD.

## Key Points

**Question:**

Does the objective classification of neuropathologic subtypes of Alzheimer disease underlie variability in the accumulation of neurofibrillary tangles and loss of neurons in the nucleus basalis of Meynert?

**Findings:**

This cross-sectional study of 1464 human brains found the fewest neurons in the nucleus basalis of Meynert in hippocampal sparing Alzheimer disease and greater accumulation of neurofibrillary tangle pathology, twice that observed in limbic predominant Alzheimer disease. Younger age at onset of cognitive symptoms was associated with greater accumulation of neurofibrillary tangles in hippocampal sparing and typical but not limbic predominant Alzheimer disease.

**Meaning:**

These results help to characterize differential involvement of the nucleus basalis of Meynert among neuropathologic Alzheimer disease subtypes, which may contribute to the differential benefit of acetylcholinesterase inhibitor treatment, especially in patients with young-onset Alzheimer disease.

## Teaser

This cross-sectional study using tissue from the Florida Autopsied Multi-Ethnic cohort assesses whether an association exists between neuropathologic subtypes of Alzheimer disease and neurofibrillary tangle accumulation or neuron loss in the nucleus basalis of Meynert, a major source of brain cholinergic innervation.

## Introduction

Alzheimer disease (AD) is a devastating neurodegenerative disorder neuropathologically characterized by abnormal tau accumulation in neurofibrillary tangles (NFTs) and the presence of extracellular amyloid-β plaque deposits.^1^ Postmortem studies of AD and more recent neuroimaging studies provide evidence that involvement of the nucleus basalis of Meynert (nbM) may be critical and early in the molecular cascade of events.^2,3,4,5,6,7^ The accumulation of NFTs in the nbM may precede entorhinal cortex and locus coeruleus involvement, making the nbM potentially one of the earliest sites where NFT accumulation occurs.^4,6,8^

We have previously shown 3 distinct regional patterns of corticolimbic NFT accumulation in subtypes of AD.^9,10,11^ Hippocampal sparing (HpSp) AD has a relatively spared hippocampus compared with the greater NFT accumulation in the cortex. Limbic predominant AD has a severely involved hippocampus compared with a relatively spared cortex. Lying between these 2 extreme AD subtypes, typical AD has the expected hippocampal and cortical NFT accumulation based on the widely accepted staging scheme proposed by Braak and Braak.^12^ In addition to the neuropathologic distinctions, these AD subtypes have striking differences in demographics and clinical progression.

Given the significance of the nbM for targeted cholinergic therapy, we sought to test the hypothesis that clinicopathologic heterogeneity of AD subtypes underlies the variability of NFT accumulation and neuronal loss in the nbM.^5,13^ Our primary goal was to investigate selective vulnerability of the cholinergic system in AD by examining the severity of NFT accumulation and neuronal loss in the nbM among AD subtypes. Our secondary goal was to evaluate whether any associations exist between NFT accumulation in the nbM and demographic or clinicopathologic changes among AD subtypes.

## Methods

### Study Samples

The Florida Autopsied Multi-Ethnic (FLAME) cohort, ^14,15^ which had been accessioned from 1991 to 2015, is derived from a consecutive series of patients who elected to participate in a deeded autopsy program via memory disorder clinic referral services. These services included community-based educational seminars for caregivers of patients with dementia and Alzheimer Association educational support groups. The FLAME cohort, which comprised 2809 individuals (1436 [51%] males and 1373 [49%] females), with an age at death ranging between 36 and 104 years, was queried for neuropathologically diagnosed AD cases and normal controls who were nondemented. We excluded 1084 study brains that were not neuropathologically diagnosed as having AD; 124 AD cases with hippocampal sclerosis because it interfered with subtype classification; 101 AD cases lacking NFT data because these could not be subtyped; and 18 AD cases with known genetic mutations. A total of 1361 AD cases remained and were termed the FLAME-AD cohort. Of the remaining 121 controls who were nondemented (Braak tangle stage<IV that lacked significant neurodegenerative pathology) that we identified for the demographic and clinicopathologic comparisons given in Table 1, we excluded 18 that were younger than the youngest FLAME-AD case (<54 years). Thus, our final sample size for controls was 103. Of the 1464 AD cases and controls, 113 were excluded from NFT density analyses and 390 were excluded from neuronal density analyses. All brains were acquired with appropriate ethical approval, and the research performed on postmortem samples was approved by the Mayo Clinic Research Executive Committee.

**Table 1.** Demographic and Clinicopathologic Characteristics by Control and AD Subtype

Characteristic	Median (IQR)				AD-Specific *P* Value^a^
	Normal Controls (n = 103)	AD Neuropathologic Subtype (n = 1361)			
		HpSp (n = 175)	Typical (n = 1014)	Limbic Predominant (n = 172)	
Female, % total of AD type, No./total No. (%)	47/103 (46)	62/175 (35)	545/1014 (54)	121/172 (70)	<.001
Educational level, y	16 (14 to 16)	16 (12 to 16)	14 (12 to 16)	14 (12 to 16)	.007
*APOE* ε4, No./total No. (%)	8/21 (38)	64/140 (46)	488/767 (64)	93/129 (72)	<.001
Clinical findings					
Age at onset, y	NA	65 (56 to 72)	71 (65 to 77)	78 (72 to 81)	<.001
Disease duration, y	NA	9 (7 to 10)	9 (6 to 12)	9 (7 to 12)	.16
Atypical presentation, No./total No. (%)	NA	57/150 (38)	89/819 (11)	3/139 (2)	<.001
MMSE					
Final score, points	27 (27 to 28)	7 (5 to 15)	13 (7 to 19)	18 (8 to 21)	.01
Change in MMSE, points, y^b^	0 (0 to 0)	–4 (–4 to –3)	–2 (–2 to –1)	–1 (–2 to –1)	<.001
Postmortem findings					
Age at death, y	73 (60 to 80)	72 (66 to 80)	81 (76 to 86)	86 (82 to 90)	<.001
Brain weight, g	1240 (1123 to 1338)	1042 (960 to 1145)	1040 (940 to 1140)	1040 (950 to 1120)	.40
Braak tangle stage	I (0 to III)	VI (V to VI)	VI (V to VI)	VI (V to VI)	<.001
Thal amyloid phase	0 (0 to 2)	5 (5 to 5)	5 (5 to 5)	5 (5 to 5)	.67
Lewy body disease, No./total No. (%)	0/103 (0)	25/175 (14)	265/1014 (26)	44/172 (26)	.003
nbM					
NFT density, per 0.125 mm^2^	1 (0 to 1)	14 (9 to 20)	10 (5 to 16)	8 (5 to 11)	<.001
Neuronal density, per mm^2^	34 (30 to 39)	22 (17 to 28)	25 (19 to 30)	26 (19 to 32)	.002

Abbreviations: AD, Alzheimer disease; *APOE* ε4, the ε4 allele of the apolipoprotein E gene; HpSp, hippocampal sparing; IQR, interquartile range; MMSE, Mini-Mental State Examination; nbM, nucleus basalis of Meynert; NA, not applicable; NFT, neurofibrillary tangle.

^a^ Normal controls were not included in Kruskal-Wallis rank sum test; thus, *P* values specifically reflect groupwise comparisons.

^b^ Estimated using a mixed linear regression model accounting for interaction of time from test to death and AD subtype.

### Neuropathologic Procedures

Standardized neuropathologic examination was performed by a single board-certified neuropathologist (D.W.D.) using the Dickson sampling scheme for neurodegenerative-centric brain dissection.^15^ To optimize sampling of the nbM at the time of brain cutting, the fixed hemibrain was cut into coronal slabs using 3 points to define the plane of section: the anterior commissure, infundibulum, and uncus. Formalin-fixed, paraffin-embedded tissue sections were cut to be 5 μm thick and mounted on to glass slides. An nbM tissue section was stained with thioflavin S and another with hematoxylin-eosin. The topographic distributions of both NFTs and amyloid-β plaques were assessed using thioflavin S immunofluorescence with an Olympus BH2 fluorescence microscope to assign Braak tangle stage^12^ and Thal amyloid phase.^16^ The Braak tangle stage ranged from 0 to III for controls and from IV to VI for AD cases. The NFT density is reported as counts per 0.125-mm^2^ microscopic field (×40 objective). Corticolimbic patterns of NFTs were examined using an AD subtype algorithm (eFigure 1 in the Supplement),^9^ which assigns an AD subtype of HpSp (175 [13%]), typical (1014 [74%]), or limbic predominant (172 [13%]). The algorithm specifically assesses the association between the hippocampus (Cornu Ammonis 1, more commonly known as CA1, and the subiculum) and the association cortices (frontal, parietal, and temporal). The α-synuclein antibody, nonamyloid-β protein component of AD amyloid, was used to assess the distribution of Lewy body pathology and classify as Lewy body disease (1:3000 dilution, rabbit, amino acids 98-115, with a cysteine residue at its C-terminus).^17^

### Neuropathologic Assessment of the nbM: NFT and Neuronal Loss Quantification

**Figure 1.**
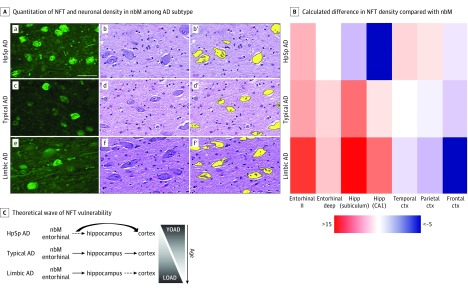
Selective Vulnerability of the Nucleus Basalis of Meynert (nbM) and Corticolimbic Structures to Neurofibrillary Tangles (NFTs) Among Neuropathologic Subtypes of Alzheimer Disease (AD) A. Thioflavin S microscopy (a, c, and e) shows greater NFT accumulation in the nbM of hippocampal sparing (HpSp) AD (a) compared with typical AD (c) and limbic predominant (limbic) AD (e). Hematoxylin-eosin–stained sections of the nbM (b, d, and f) were digitally quantified (b′, d′, and f′, respectively). Fewer neurons are observed in HpSp AD (b) compared with typical AD (d) and limbic predominant AD (f). Scale bar represents 50 μm. B. Heatmap of differences calculated between brain region of interest and the nbM, as exampled by the more severe involvement of the entorhinal cortex compared with the nbM in limbic predominant AD, shown in warmer colors, and the less severe involvement of the hippocampus (Hipp) in HpSp AD compared with the nbM, shown in cooler colors. C. We hypothesize that, although both the nbM and entorhinal cortex (ctx) are involved early among AD subtypes and across aging, the cortex may be more vulnerable in HpSp AD. By contrast, the pattern of greater vulnerability of limbic structures is manifested in both limbic predominant AD and perhaps as a function of older age. LOAD indicates late-onset AD; YOAD, young-onset AD.

The term *nucleus basalis* includes all neuronal components of the nbM, of which more than 90% are magnocellular neurons that are cholinergic.^18^ At the time of neuropathologic examination, thioflavin S microscopy was used to quantify NFT counts in the nbM (Figure 1). The prospective assessment of NFT density (NFT count per 0.125 mm^2^) was performed by D.W.D., who was blinded to AD subtype algorithm classification. An Olympus BH2 fluorescence microscope was used to evaluate greatest lesion density at low magnification. Subsequently, a ×40 objective was used for 2 or more microscopic fields to count the area of greatest density. A detailed overview of sample size by neuroanatomic level, as well as information regarding cases excluded, is given in eTables 1 and 2 in the Supplement.

To quantify the neuronal density of the nbM among a large series of AD cases and controls, we implemented high-throughput digitization of hematoxylin-eosin–stained slides (Figure 1). Detailed digital pathology methods and neuroanatomic assessment can be found in eAppendix 1 in the Supplement. The level of the nbM was neuroanatomically classified based on the anterior-to-posterior extent of the nucleus (eFigure 2 in the Supplement).^19^ Given the lack of discrete boundaries of the nbM with neighboring cell groups,^20^ we implemented specific neuroanatomic boundaries that enabled us to systematically capture the neuronal density of nbM neurons. Our application of neuroanatomic boundaries and assessment of the anterior-to-posterior extent of the nbM was informed by Mesulam and Geula^19^ and the recently revisited anatomic assessment of the nbM by Liu et al.^21^ We used the anterior commissure, globus pallidus, fornix, and mammillary body to facilitate identification of nbM level.^18,19,21^ The slides with hematoxylin-eosin–stained tissue were annotated, being blinded to both disease status (control vs AD) and AD subtype, using ImageScope software (Leica Biosystems). The annotated nbM was then batch analyzed in Aperio eSlide Manager (Leica Biosystems) using a custom-designed digital pathology macro to identify surviving neurons in the nbM on hematoxylin-eosin–stained sections. The macro was built to recognize the well-circumscribed, basophilic properties of the nbM neuron, as shown in Figure 1. The data are exported as counts, which were divided by the area annotated. Neuronal density is reported as neurons per millimeters squared, not total number of neurons. The mean size of objects counted was additionally exported to examine neuronal shrinkage (eAppendix 2 in the Supplement). Data provided in the present study were derived from the anterior nbM (eFigure 1 in the Supplement) because this level contains the most widespread and noticeable portion of the nbM^3,19^ and was the most robustly sampled (eTables 1 and 2 in the Supplement). As shown in eFigure 2 in the Supplement, the cholinergic neurons at the anterior level correspond to the Ch4am, Ch4al, and Ch4ai subsectors of the nbM.^18,19,21^

### Clinical History

In this cross-sectional study, clinical history was abstracted from existing clinical records made available by brain bank participants or family members, as previously described.^14^ The neurologic summary or brain bank questionnaire was reviewed for details relating to the age when the first cognitive symptoms began. The date of birth was subtracted from the approximate date at onset to identify the age at onset in years. The date at onset was subtracted from date of death to identify the disease duration in years. Atypical clinical presentations were recorded that differed from the expected amnestic presentation more commonly observed among patients having a clinical diagnosis of AD, such as primary progressive aphasia, frontotemporal dementia, posterior cortical atrophy, and corticobasal syndrome.^14,22,23^ Any available Mini-Mental State Examination (MMSE)^24^ score (range, 0-30) and date of test were recorded. The MMSE scores obtained within 3 years of death were recorded as the final MMSE score, serving as a measure of cognitive impairment. Three or more MMSE scores were required to estimate cognitive decline measured as a change in MMSE (eAppendix 1 in the Supplement). The first and last MMSE test dates were required to be more than 1 year apart.

### Statistical Analysis

Continuous variables (eg, age at onset, nbM NFT density) are represented using medians and interquartile ranges. Categorical variables (eg, sex) were summarized using frequencies and percentages. The Kruskal-Wallis rank sum test was used to test for differences in continuous measures, while the Pearson χ^2^ test was used to compare proportions among AD subtype groups. The associations of demographic (sex, educational level, and the apolipoprotein E [*APOE*] ε4 carrier status) and clinical variables (age at onset, disease duration, and final MMSE score) with NFT accumulation in the nbM among AD subtypes was examined using 3 multivariable linear regression models that were created to investigate within-subtype differences. Three additional models were used to investigate neuronal density. All tests were 2-sided, and *P* < .05 was considered statistically significant. All statistical analyses were performed using R statistical software, version 3.4.2 (R Foundation for Statistical Computing) and completed January 2019.

## Results

### Demographic, Clinicopathologic, and Neuropathologic Differences Among AD Subtypes

Table 1 provides the demographic, clinical, and neuropathologic findings among 1361 AD subtypes and 103 nondemented controls included for comparison. The HpSp AD cases were more commonly observed in men (113 [65%]) compared with typical AD (469 [46%]) and limbic predominant AD (51 [30%]) (*P* < .001). Individuals with HpSp AD had a higher level of education (median, 16 years; interquartile range [IQR], 12-16 years; *P* = .007) and lowest frequency of the *APOE* ε4 risk variant (64 of 140 [46%]; *P* < .001). Among clinical findings, HpSp AD cases were the youngest to present with cognitive symptoms (median, 65 years; IQR, 56-72 years) compared with typical AD cases (median, 71 years; IQR, 65-77 years) and limbic predominant AD cases (median, 78 years; IQR, 72-81 years) (*P* < .001). The proportion of cases with an atypical clinical presentation was highest in HpSp AD cases (57 of 150 [38%]), lower in typical AD (89 of 819 [11%]), and lowest in limbic predominant AD (3 of 139 [2%]) (*P* < .001). The final median (IQR) MMSE score was lowest in HpSp AD (7; 5-15 points), higher in typical AD (13; 7-19 points), and highest in limbic predominant AD (18; 8-21 points) (*P* = .01). Moreover, the change in median MMSE over time was faster in HpSp AD cases (4 points lost per year; IQR, −4 to −3 points) compared with both typical AD (2 points lost per year; IQR, −2 to −1 points) and limbic predominant AD (1 point lost per year; IQR, −2 to −1 points) (*P* < .001). Using linear regression modeling to adjust for age at death and sex, AD subtype differences remained significant, with HpSp AD cases estimated to decline the fastest at 5 points lost per year (IQR, −6 to −4 points; *P* < .001).

In addition to observed demographic and clinical differences, there were major differences in neuropathologic characteristics among AD subtypes (Table 1). The HpSp AD cases were the youngest at death (median, 72 years; IQR, 66-80 years) compared with typical AD (median, 81 years; IQR, 76-86 years), who were younger than limbic predominant AD (median, 86 years; IQR, 82-90 years) (*P* < .001). Braak tangle stage differed at the group level (*P* < .001); however, between-group differences were less evident because the median (IQR) stage was the same for each AD subtype (VI; V-VI). The presence of coexisting Lewy body disease was lower in HpSp AD (25 [14%]) compared with typical AD (265 [26%]) and limbic predominant AD (44 [26%]) (*P* = .003).

### Demographic and Clinical Associations With NFT Accumulation and Neuronal Density in the nbM Stratified by AD Subtype

**Figure 2.**
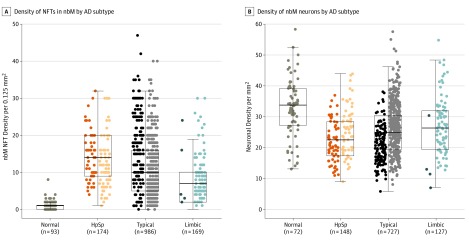
Differences in Neurofibrillary Tangle (NFT) and Neuronal Density in the Nucleus Basalis of Meynert (nbM) Among Alzheimer Disease (AD) Subtypes Data are displayed as jitter plots overlaying box plots of the 25th to 75th percentile, with the middle horizontal line representing the median. Nondemented normal controls are displayed for reference. Within each AD subtype, individuals younger than 65 years of age presenting with cognitive problems are displayed on the left and individuals 65 years or older are displayed on the right (lighter color). A, The NFT density in the nbM was measured using thioflavin S fluorescence microscopy. HpSp AD cases have the greatest accumulation of NFTs compared with typical AD, which is greater than limbic predominant (limbic) AD. B, Neuronal density was measured using a custom-designed digital pathology macro on hematoxylin-eosin–stained sections of the nbM. Hippocampal sparing (HpSp) AD has fewer remaining neurons compared with typical AD, which has fewer compared with limbic predominant AD. Pairwise comparisons were performed using Mann-Whitney rank sum test.

The NFT accumulation (count per 0.125 mm^2^) in the anterior level of the nbM was highest in 163 HpSp AD cases (median, 14; IQR, 9-20), lower in 937 typical AD cases (median, 10; IQR, 5-16), and lowest in 163 limbic predominant AD (median 8; IQR, 5-11) (*P* < .001) (Figure 2A; Table 1). Neuronal density (per millimeter squared) in the nbM was lowest in 148 HpSp AD cases (median, 22; IQR, 17-28) compared with 727 typical AD (median, 25; IQR, 19-30) and 127 limbic predominant AD (median, 26; IQR, 19-32) (*P* = .002) (Figure 2B). To test that the difference in nbM NFT density and neuronal density did not simply reflect differences observed in cognitive impairment among individuals with the various AD subtypes, we used multivariable linear regression models to adjust for final MMSE score. Group differences remained among AD subtypes for nbM NFT density (*P* < .001) and nbM neuronal density (*P* = .001). To put in perspective the relative difference in NFT accumulation in areas routinely assessed in Braak tangle staging compared with the nbM, Figure 1B shows a heatmap of calculated differences between brain regions of interest and the nbM. This is exampled by the more severe involvement of the hippocampus compared with the nbM in limbic predominant AD, as shown by warmer colors, and the less severe involvement of the hippocampus compared with the nbM in HpSp AD, as shown by cooler colors. The calculated differences are given in eFigure 3 in the Supplement.

**Figure 3.**
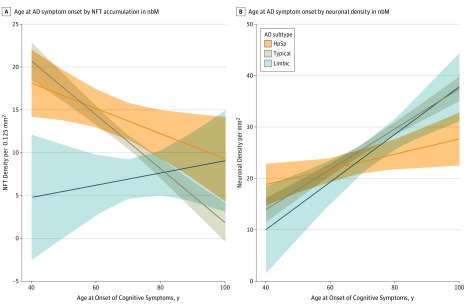
Association Between Age at Onset of Cognitive Symptoms and Neurofibrillary Tangle (NFT) Density and Neuronal Density in the Nucleus Basalis of Meynert (nmB) Among Alzheimer Disease (AD) Subtypes Best-fit lines represent the association of age at onset per AD subtype while adjusting for other covariates in the regression models found in Table 2. A, Younger age at onset of cognitive symptoms is significantly associated with greater NFT accumulation measured in the nbM of hippocampal sparing (HpSp) AD and typical AD but not limbic predominant (limbic) AD. B, The association between age at onset of cognitive symptoms and neuronal density measured in hematoxylin-eosin–stained sections of the nbM among AD subtypes is significant among all of the AD subtypes.

To examine the overlap in NFT accumulation and neuronal density differences observed among AD subtypes in Figure 2, we performed multivariable regression analyses within each AD subtype for a total of 6 models. Table 2 summarizes the expected change in NFT accumulation and neuronal density in the nbM, adjusted for demographic and clinical variables of interest and their associations within the AD subtype. Younger age at onset of cognitive symptoms was significantly associated with higher NFT counts in the nbM of HpSp AD (*P* < .001) (Figure 3A). Thus, for every 10 years younger age at onset, the number of NFTs was expected to be higher by 1.5 (95% CI, −2.9 to −0.15; *P* = .03) in HpSp AD cases and by 3.2 (95% CI, −3.9 to −2.4; *P* < .001) in typical AD cases. In addition, within the typical AD cases, females were expected to have 2.5 (95% CI, 1.4-3.5) more NFTs than males (*P* < .001) and *APOE* ε4 carriers to have 1.3 (95% CI, 0.15-2.5) more NFTs than *APOE* ε4 noncarriers (*P* = .03). For every 10-point decrease in final MMSE of typical AD cases, the number of nbM NFTs was expected to increase by 1.8 (95% CI, −3.2 to −0.31; *P* = .02). Accumulation of NFTs in the nbM of limbic predominant AD cases was not associated with the observed demographic and clinical variables. Regression analyses of neuronal density largely reflected the same pattern of associations with demographic and clinical variables observed with NFT accumulation in HpSp AD and typical AD (Table 2, eAppendix 2 in the Supplement). However, new associations with neuronal density emerged in limbic predominant AD. For every 10 years’ younger age at onset, the number of neurons was expected to be lower by 4.6 (95% CI, 2.3-7.0) in limbic predominant AD cases (*P* < .001) (Figure 3B). In addition, limbic predominant cases were observed to have 4.3 fewer neurons (95% CI, 0.47-8.1) for every 10-point decrease in MMSE.

**Table 2.** Regression Analyses of Demographic and Clinical Variables Estimating NFT Accumulation and Neuronal Density in the nbM Stratified by AD Subtype

Variable^a^	HpSp AD		Typical AD		Limbic Predominant AD	
	β (95% CI)	*P* Value	β (95% CI)	*P* Value	β (95% CI)	*P* Value
Dependent variable: nbM NFT density^b^						
Female sex^c^	0.91 (–1.7 to 3.6)	.50	2.5 (1.4 to 3.5)	<.001	1.1 (–1.0 to 3.2)	.32
Educational level, 1 y	–0.24 (–0.75 to 0.27)	.35	–0.01 (–0.25 to 0.22)	.91	–0.30 (–0.73 to 0.12)	.16
*APOE* ε4^c^	1.3 (–1.3 to 3.9)	.32	1.3 (0.15 to 2.5)	.03	–0.33 (–2.7 to 2.1)	.79
Age at onset, 10 y	–1.5 (–2.9 to –0.15)	.03	–3.2 (–3.9 to –2.4)	<.001	0.61 (–1.1 to 2.3)	.48
Disease duration, 1 y	–0.14 (–0.59 to 0.32)	.56	0.040 (–0.11 to 0.19)	.57	0.090 (–0.21 to 0.39)	.57
Last MMSE score, 10 points	–0.89 (–4.1 to 2.3)	.59	–1.8 (–3.2 to –0.31)	.02	–0.050 (–2.9 to 2.8)	.97
Dependent variable: nbM neuronal density						
Female sex^c^	1.1 (–1.6 to 3.8)	.42	–1.1 (–2.3 to –0.010)	.048	–1.2 (–4.2 to 1.8)	.45
Educational level, 1 y	0.25 (–0.25 to 0.75)	.33	0.10 (–0.15 to 0.34)	.44	0.00 (–0.56 to 0.56)	.99
*APOE* ε4^c^	–0.26 (–2.9 to 2.3)	.84	–0.13 (–1.4 to 1.1)	.84	–2.0 (–5.3 to 1.2)	.22
Age at onset, 10 y	1.5 (0.13 to 2.8)	.03	4.0 (3.2 to 4.7)	<.001	4.6 (2.3 to 7.0)	<.001
Disease duration, 1 y	–0.44 (–0.89 to 0.02)	.06	0.11 (–0.040 to 0.26)	.17	0.23 (–0.18 to 0.64)	.27
Last MMSE score, 10 points	–0.49 (–3.7 to 2.8)	.77	1.4 (–0.030 to 2.9)	.06	4.3 (0.47 to 8.1)	.03

Abbreviations: AD, Alzheimer disease; *APOE* e4, the ε4 allele of the apolipoprotein E gene; HpSp, hippocampal sparing; MMSE, Mini-Mental State Examination; nbM, nucleus basalis of Meynert; NFT, neurofibrillary tangle.

^a^ The unit for each continuous variable is indicated alongside the variable.

^b^ Data are presented as the estimated change in the number of neurofibrillary tangles per unit change of the variable of interest in the 3 models.

^c^ Female sex and the presence of the ε4 risk allele are discrete variables set at 1 and can be interpreted as associating positively if the outcome is positive.

## Discussion

In this retrospective study of patients who were derived from memory disorder clinics for diagnosis and treatment of neurocognitive disorders and who ultimately came to autopsy, our data support the novel concept that there is an association between the severity of NFT pathology in the nbM and corticolimbic patterns of NFT pathology in the brain. In the FLAME-AD cohort, the nbM was more vulnerable to neuropathologic insult in HpSp AD cases compared with typical AD, which were more vulnerable than limbic predominant AD and nondemented normal controls. Younger age at onset was associated with greater NFT accumulation in the nbM of HpSp AD and typical AD but not limbic predominant AD. However, we did observe fewer nbM neurons remaining in limbic predominant cases, suggesting perhaps a non-NFT mediated association.

The nbM is one of the most vulnerable brain regions to NFT pathology in AD.^3,13^ On the basis of the importance of the nbM for targeted treatment by acetylcholinesterase inhibitor therapies, we sought to test the hypothesis that the cholinergic system is differentially involved among AD subtypes. Inherent to autopsy studies, investigations are often performed at the end stage of the disease. Evidence suggests that 80% to 88% of the cholinergic neurons in the posterior nbM are depleted in AD compared with 29% to 54% in the anterior nbM.^19^ Data from the current study were derived from the anterior nbM, which facilitates investigation of clinicopathologic contributors to variability in NFT accumulation and in nbM neuronal loss prior to the burnout observed in the posterior nbM of AD brains. We found twice the number of NFTs per microscopic field in the nbM of HpSp AD cases compared with limbic predominant AD cases. This finding emphasizes the association of the NFT pathology in the anterior nbM to cortical, rather than limbic, location. Evidence supports that cholinergic projections to the hippocampus are derived from cholinergic neurons in the medial septal nucleus (Ch1) and vertical nucleus of the diagonal band (Ch2) of the basal forebrain.^18,20,21^ However, cell loss is not particularly evident in these hippocampal projecting nuclei in comparison with cell shrinkage of cholinergic neurons.^25^ Retrograde transport of neurotrophic factors from target regions is hypothesized to account for the cell shrinkage along the rostrocaudal extent of basal forebrain structures.^25,26^ This process elicits an intriguing hypothesis in HpSp AD in which cortical vulnerability to NFTs is a result of diminished cholinergic innervation from the nbM, which, via a feedback loop, contributes to exacerbation of nbM vulnerability to NFTs through a reduction of neurotrophic support from the cortex.

We found the fewest neurons in the nbM of HpSp AD cases compared with typical AD and limbic predominant AD, which were all lower than the nbM neuronal counts of controls. As expected, an inverse association was observed between NFT accumulation and neuronal density in the nbM.^3^ There is evidence to suggest that neuronal loss in the nbM precedes that in the locus coeruleus or entorhinal cortex,^6^ 2 areas that are hypothesized to be initiation sites for NFT accumulation.^8,12^ Although we could not compare the rate of NFT accumulation in postmortem tissue, we provided data on differential patterns in the entorhinal cortex, hippocampus, and association cortices relative to the nbM among AD subtypes. These observations are especially interesting when taken together with previous findings of more severe NFT accumulation in the cortex of younger-onset AD cases compared with potentially greater vulnerability of the hippocampus in late-onset AD cases.^9,10,11,14,27,28,29^ We hypothesize that upstream factors, likely genetic, contribute both to the corticolimbic pattern of NFT vulnerability among AD subtypes and between young-onset AD and late-onset AD. We observed a wave of vulnerability in which the exacerbation of nbM NFTs in HpSp AD may leave the cortex more vulnerable to NFT accumulation, perhaps via a biologically accelerated process or through a mechanism of disinhibition. By contrast, the limbic predominant AD cases had an exacerbation of areas vulnerable early in the Braak-like pattern of NFT accumulation, perhaps via a biologically restrictive process that relatively confines pathology to limbic areas.

Given the significant demographic and clinicopathologic differences among AD subtypes, we elected to perform an analysis with separate covariate effects by AD subtype to enhance our ability to detect meaningful associations not diluted by the contribution of AD as a whole. We observed greater NFT accumulation associated with younger age at onset in HpSp AD and typical AD but not limbic predominant AD. Our data support a neurochemical study that has shown more severe cholinergic deficits in the brains of younger decedents (died <79 years of age) compared with older decedents (≥80 years of age), which suggests NFT accumulation in the nbM may underlie more widespread pathology and likely more severe cholinergic deficits.^30^ This outcome is of particular interest given that age is the strongest risk factor for AD dementia, yet we and others have shown that individuals with young-onset AD may paradoxically represent a more aggressive form of the disease.^14,31,32^ Given the focused association of age at onset in HpSp AD cases to the exclusion of other demographic and clinical variables and lack of a similar finding in the limbic predominant AD cases, future studies may seek to identify as-of-yet unknown contributors that may be unique in each of these extreme AD subtypes. It will be of particular interest to examine non–*APOE* ε4-associated genetic mechanisms that affect gene transcription, which have been shown to influence the temporal course of nbM involvement in AD.^33^

We observed significant differences in NFT accumulation in the nbM among *APOE* ε4 carriers and noncarriers in typical AD, which is consistent with previous findings showing subtle differences in response to cholinesterase inhibitors based on *APOE* ε4 carrier status.^34^ However, this finding is inconsistent with a previous immunohistochemical study that did not detect differences between *APOE* ε4 carriers and noncarriers using the Alz-50 antibody.^35^ This inconsistency could be the result of differences in detection methods of NFT pathology, or the finding may be specific to typical AD cases and not readily detected in a cohort also including the extreme subtypes, that is, HpSp AD and limbic predominant AD. Our study provides additional evidence that female sex was associated with selective vulnerability of the nbM to NFTs in typical AD. These findings are consistent with those reported in previous studies showing that women have greater amounts of global AD pathology, especially greater numbers of NFTs than men.^14,36,37^ Moreover, our results support the immunohistochemical study of the nbM that has shown greater tau immunoreactivity in women than in men.^35^

### Limitations

The results of the current study should be informative for clinicians diagnosing memory disorders and treating patients with such disorders. However, it is important to acknowledge various biases associated with consenting to autopsy, which can include self-selection, higher educational level, marital status, and race/ethnicity.^38^ Moreover, longitudinal data relating to cognitive decline measured using MMSE was limited to a subset of cases. Given the size of our autopsied AD cohort, we did not use stereologic methods to assess the volume of the nbM. To offset this limitation in our study design, we used digital pathology measures to objectively quantify neuronal density of the anterior nbM with neuroanatomic boundaries defined by neighboring structures.

## Conclusions

The findings reported in the present study, as well as those reported by others, underscore the importance of considering age at onset, sex, and *APOE* genotype when interpreting outcomes in AD.^9,14,29,32,36^ Our future studies will expand on these findings in a longitudinally followed cohort to extend our understanding of the differential treatments^39^ administered to individuals with HpSp AD, typical AD, and limbic predominant AD.

## Notes

Supplement. **eAppendix 1.** Methods **eAppendix 2.** Results **eFigure 1.** Alzheimer Disease’s (AD) Subtyping Algorithm Classification **eFigure 2.** The Anterior-to-Posterior Extent of the Nucleus Basalis of Meynert **eFigure 3.** Relative Difference in NFT Accumulation in the nbM Compared to Areas Involved Early (eg, Entorhinal) and Later (eg, Association Cortex) in the Disease Progression According to Braak Tangle Staging **eTable 1.** Sample Size Inclusion and Exclusion From Analyses Investigating Neurofibrillary Tangle Differences in the Nucleus Basalis of Meynert Within the Total Cohort of Normal Controls and Alzheimer’s Disease Cases or by AD Subtype **eTable 2.** Sample Size Inclusion and Exclusion From Analyses Investigating Neuronal Differences in the Nucleus Basalis of Meynert Within the Total Cohort of Normal Controls and Alzheimer’s Disease Cases or by AD Subtype **eReferences**
