# Supplementary material for: Selective Vulnerability of the Nucleus Basalis of Meynert Among Neuropathologic Subtypes of Alzheimer Disease
Source: JAMA Neurol. 2019 Oct 28;77(2):225–33. doi: 10.1001/jamaneurol.2019.3606 (PMC6820048; doi:10.1001/jamaneurol.2019.3606)
Supplement: Supplement. — eAppendix 1. Methods eAppendix 2. Results eFigure 1. Alzheimer Disease’s (AD) Subtyping Algorithm Classification eFigure 2. The Anterior-to-Posterior Extent of the Nucleus Basalis of Meynert eFigure 3. Relative Difference in NFT Accumulation in the nbM Compared to Areas Involved Early (eg, Entorhinal) and Later (eg, Association Cortex) in the Disease Progression According to Braak Tangle Staging eTable 1. Sample Size Inclusion and Exclusion From Analyses Investigating Neurofibrillary Tangle Differences in the Nucleus Basalis of Meynert Within the Total Cohort of Normal Controls and Alzheimer’s Disease Cases or by AD Subtype eTable 2. Sample Size Inclusion and Exclusion From Analyses Investigating Neuronal Differences in the Nucleus Basalis of Meynert Within the Total Cohort of Normal Controls and Alzheimer’s Disease Cases or by AD Subtype eReferences [file jamaneurol-77-225-s001.pdf]

Supplementary Online Content

Hanna Al-Shaikh FS, Duara R, Crook JE, et al. Selective vulnerability of the nucleus basalis of Meynert among neuropathologic subtypes of Alzheimer disease. *JAMA Neurol*. Published online October 28, 2019. doi:10.1001/jamaneurol.2019.3606

eAppendix 1. Methods

eAppendix 2. Results

eFigure 1. Alzheimer's Disease (AD) Subtyping Algorithm Classification

eFigure 2. The Anterior-to-Posterior Extent of the Nucleus Basalis of Meynert

eFigure 3. Relative Difference in NFT Accumulation in the nbM Compared to Areas Involved Early (eg, Entorhinal) and Later (eg, Association Cortex) in the Disease Progression According to Braak Tangle Staging

eTable 1. Sample Size Inclusion and Exclusion From Analyses Investigating Neurofibrillary Tangle Differences in the Nucleus Basalis of Meynert Within the Total Cohort of Normal Controls and Alzheimer's Disease Cases or by AD Subtype

eTable 2. Sample Size Inclusion and Exclusion From Analyses Investigating Neuronal Differences in the Nucleus Basalis of Meynert Within the Total Cohort of Normal Controls and Alzheimer's Disease Cases or by AD Subtype

eReferences

This supplementary material has been provided by the authors to give readers additional information about their work.

eAppendix 1. Methods

Neuropathologic assessment of neuronal loss in the nbM using digital pathology

Microscope slides were digitized using the Aperio AT2 for whole slide scanning (Leica Biosystems, Buffalo Grove, IL). Slides were viewed and analyzed using Aperio ImageScope (Leica Biosystems, Buffalo Grove, IL), which enables macroscopic (1x) to microscopic (20x) inspection of H&E slides. The level of the nucleus basalis of Meynert (nbM) was neuroanatomically classified based upon the anterior-to-posterior extent of the nucleus (**eFigure 2**).[1] Given the lack of discrete boundaries of the nbM with neighboring cell groups,[2] we implemented specific neuroanatomic boundaries that enabled us to systematically capture the neuronal density of nbM neurons. Our application of neuroanatomic boundaries and assessment of the anterior-to-posterior extent of the nbM was informed by Mesulam & Geula[1] and the Liu et al.'s revisited neuroanatomic assessment of the nbM.[3] We used the anterior commissure, globus pallidus, fornix, and mammillary body to facilitate identification of nbM level.[1,3,4] These structures were additionally used to operationally define nbM boundaries for tracing using Aperio ImageScope software. The superior border utilized both the inferior aspect of the anterior commissure and/or globus pallidus depending on the level. The medial border was identified to be perpendicular to the lateral border of the optic tract. The entorhinal sulcus made up the inferior border, and the junction between the putamen and globus pallidus was used to define the lateral border. These boundaries were assessed on choline acetyltransferase (ChAT, 1:500, goat, Biocare Medical, Pacheco, CA) immunostained sections in 10 of our controls to ensure the boundaries captured the majority of ChAT-positive cells in the objectively defined nbM.

Of the 103 controls, 15 (15%) were excluded from NFT density analyses as tissue was not available for review or the nbM was sampled outside of the anterior level (**eTable 1**). Of the 1361 AD cases, 98 (7%) were excluded from NFT density analyses. Of the 103 controls, 31 (30%) were excluded as tissue was not available for review or the nbM was sampled outside of the anterior level (**eTable 2**). Of the 1361 AD cases, 359 (26%) were excluded from NFT density analyses.

Estimating change in MMSE

Due to the serial nature of (Mini-Mental State Examination) MMSE data being tested at varying time points and intervals, we elected to estimate the change in MMSE per year for each case using a mixed linear model accounting for the interaction of time and AD subtype. Time was measured as the number of years from death each test was taken, and the estimates of MMSE per year were based on cases that had three or more recorded MMSE tests.

© 2019 Hanna Al-Shaikh FS et al. *JAMA Neurology*.

eAppendix 2. Results

Neuronal shrinkage differences too low to explain observed differences in nbM NFT density among AD subtypes

Our neuronal count data export additionally provided us with average neuronal size, which confirms previous findings[5] that AD cases had a smaller cell size (median: 341 μm^2 [interquartile range: 320 μm^2,367 μm^2]) compared to controls (359 μm^2 [332 μm^2,389 μm^2]) (p<0.001). Cell shrinkage was greatest in our HpSp AD cases (338 μm^2 [318 μm^2,359 μm^2]) compared to typical AD (341 μm^2 [320 μm^2,367 μm^2]) and limbic predominant AD (349 μm^2 [326 μm^2,371 μm^2]) (p=0.052). However, these differences in neuronal shrinkage among AD subtypes appear too low to explain the observed difference in nbM neuronal density among AD subtypes.

Regression modeling of nbM neuronal density

Regression analyses of neuronal density largely reflected the same pattern of relationships with demographic and clinical variables observed with NFT accumulation in HpSp AD and typical AD (**Table 2**). However, new relationships with neuronal density emerged in limbic predominant AD. Younger age at onset of cognitive symptoms was significantly associated with fewer neurons in the nbM of HpSp AD (p=0.032), typical AD cases (p<0.001), and limbic predominant AD (p<0.001). Thus, for every 10 years younger age at onset, the number of neurons was expected to be lower by 1.5 [95% CI: 0.13,2.8] in HpSp AD cases, 4.0 [95% CI: 3.2,4.7] in typical AD cases, and 4.6 [95% CI: 2.3,7.0] in limbic predominant AD cases. In HpSp AD, a longer disease duration approached significance with the number of neurons in the nbM expected to be lower by 0.44 [95% CI: -0.89,0.020] for every one year longer disease duration (p=0.058). Within the typical AD cases, females had an average of 1.1 [95% CI: -2.3,-0.010] fewer neurons than males (p=0.048). A lower MMSE score within three years of death approached significance with fewer neurons in the nbM of typical AD cases. For every 10 point decrease in MMSE, the number of neurons was expected to decrease by 1.4 [95% CI: -0.030,2.9] (p=0.055). Limbic predominant cases were observed to have on average 4.3 fewer neurons [95% CI: 0.47,8.1] for every 10 point decrease in MMSE.

© 2019 Hanna Al-Shaikh FS et al. *JAMA Neurology*.

eFigure 1. Alzheimer's Disease (AD) Subtyping Algorithm Classification. The first step of the AD classification algorithm assigns cases based upon quartile classification of the hippocampus to cortex ratio of neurofibrillary tangle density. This step is intended to prevent mild cases from being incorrectly classified as limbic predominant AD. The second step utilizes regional differences in hippocampal and cortical values to offset under- and overcalling of AD subtypes.[6]

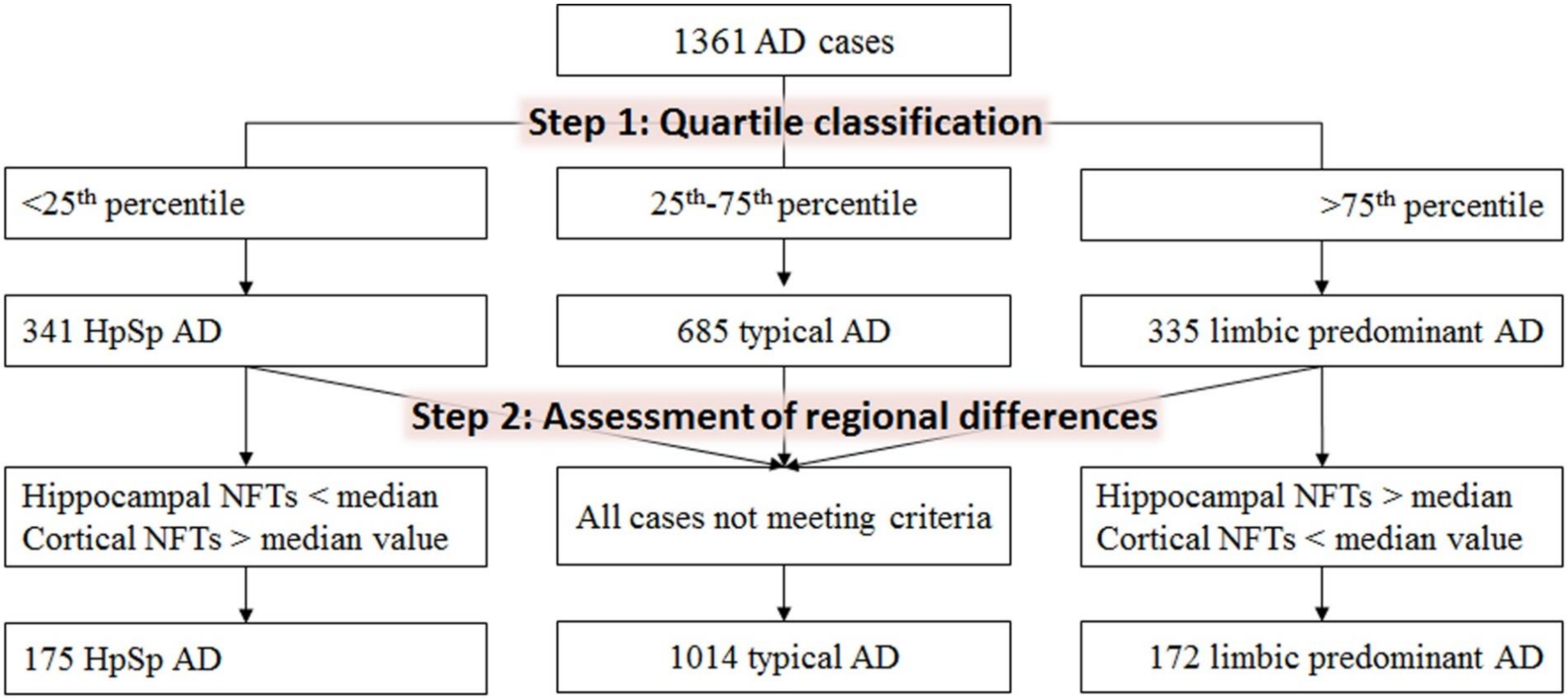

© 2019 Hanna Al-Shaikh FS et al. *JAMA Neurology*.

eFigure 2. The Anterior-to-Posterior Extent of the Nucleus Basalis of Meynert. The Dickson sampling scheme for neurodegenerative-centric brain dissection utilizes three points to define the plane of section: anterior commissure, infundibulum, and uncus. This provides an effective means for routine investigation of the nucleus basalis of Meynert (nbM) in the FLAME cohort. Guided by the work of Mesulam&Geula,[1] we assigned (**A-D**) nbM levels based upon neighboring neuroanatomic landmarks (reprinted with permission, license number 4630380158843). The current study specifically investigates (**E-G)** the anterior nucleus basalis, which includes all neuronal components. However, we provide terminology utilized in the literature in reference to cholinergic projection neurons found in Ch4 sectors for ease of comparison.[1,4,7] The anterior nbM level overlaps with the decussation of the anterior commissure,[4] which is referred to as rostral anterior and caudal anterior by Liu et al.[3] (**E**) The rostral anterior nbM was defined by the presence of the decussation of the anterior commissure that was located ventral to an unsegmented globus pallidus. (**F**) The caudal anterior nbM was defined by the split of the anterior commissure that was located ventral to an unsegmented globus pallidus. The (**G**) anterointermediate nbM was defined by the anterior commissure located ventral to the putamen, and the presence of the globus pallidus externa and globus pallidus interna. The (**H**) intermediate nbM level was defined by the presence of the mammillary body, and where the anterior commissure descends into the temporal stem. We did not find any case in the FLAME series that would be consistent with posterior nbM, which could be identified at the level of the amygdalo-hippocampal junction. The pre-anterior level, as described by Liu et al.,[3] could be found in the section anterior to the appearance of the anterior commissure where nucleus accumbens is visible. The pre-anterior level is not shown below to enable a concise figure. Acronyms: AC=anterior commissure, FLAME=FLorida Autopsied Multi-Ethnic GP=globus pallidus, GPe=globus pallidus externa, GPi=globus pallidus interna, MB=mammillary body, Put=putamen.

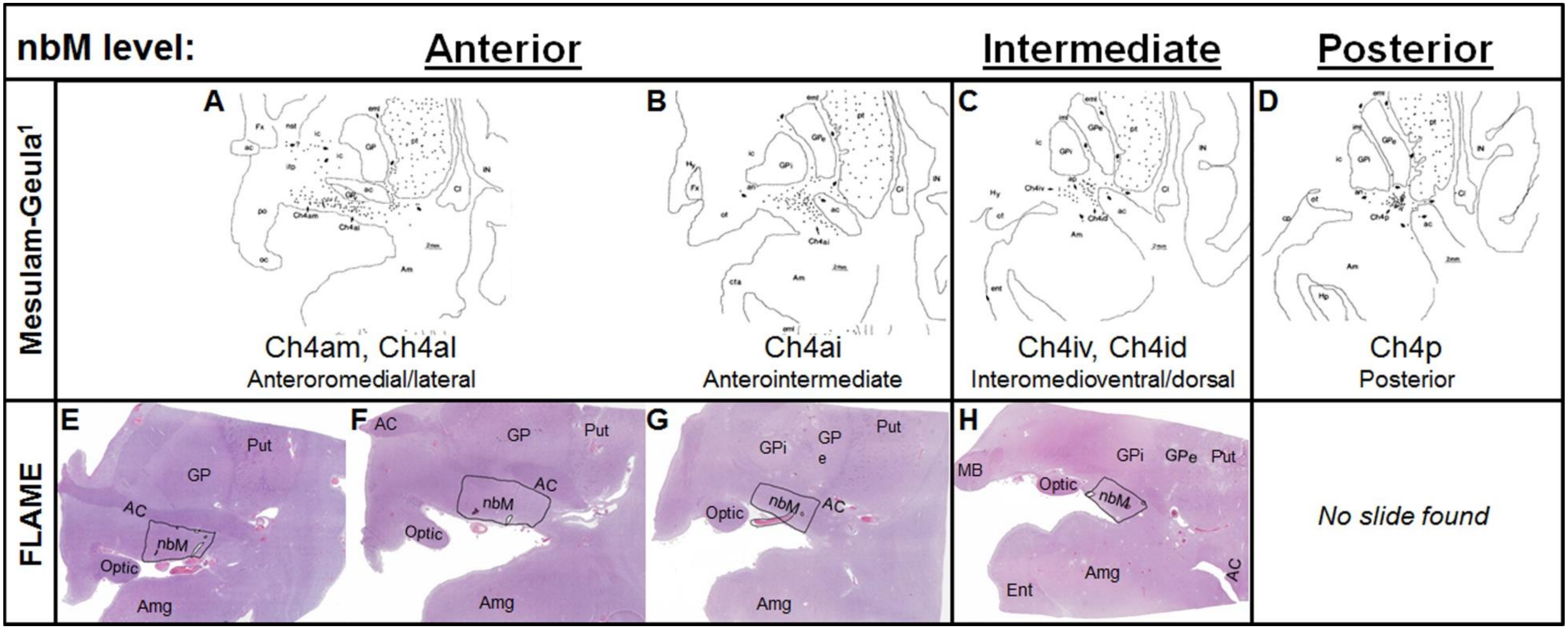

© 2019 Hanna Al-Shaikh FS et al. *JAMA Neurology*.

eFigure 3. Relative Difference in NFT Accumulation in the nbM Compared to Areas Involved Early (eg, Entorhinal) and Later (eg, Association Cortex) in the Disease Progression According to Braak Tangle Staging.[8] The nbM NFTs were subtracted from the brain region of interest with median value displayed overlaying corresponding heatmap color to provide a relative value. The entorhinal cortex layer II in HpSp AD was more involved than the nbM, translating to 4 more NFTs per 0.125 mm^2 field of view. We observed a higher number of NFTs in the entorhinal cortex layer II relative to the nbM in typical AD, and the highest relative amount in limbic predominant AD. Deeper layers of the entorhinal cortex differed to a lesser extent, but with the same pattern of increasing differences from HpSp AD < typical AD ≈ limbic predominant AD. The hippocampus in HpSp AD had far fewer NFTs than the nbM, whereas typical AD and limbic predominant AD had many more. This pattern was inverted in temporal cortex and parietal cortex where HpSp AD had more tangles in the cortex compared to the nbM. Typical AD had similar amounts in temporal cortex and parietal cortex compared to the nbM, but limbic predominant AD had far fewer. The frontal cortex was less involved compared to the nbM for each subtype. Interestingly, in controls we did not observe a difference in median NFT density in entorhinal cortex and hippocampus compared to the nbM. The cortex of controls was less affected in the nbM.

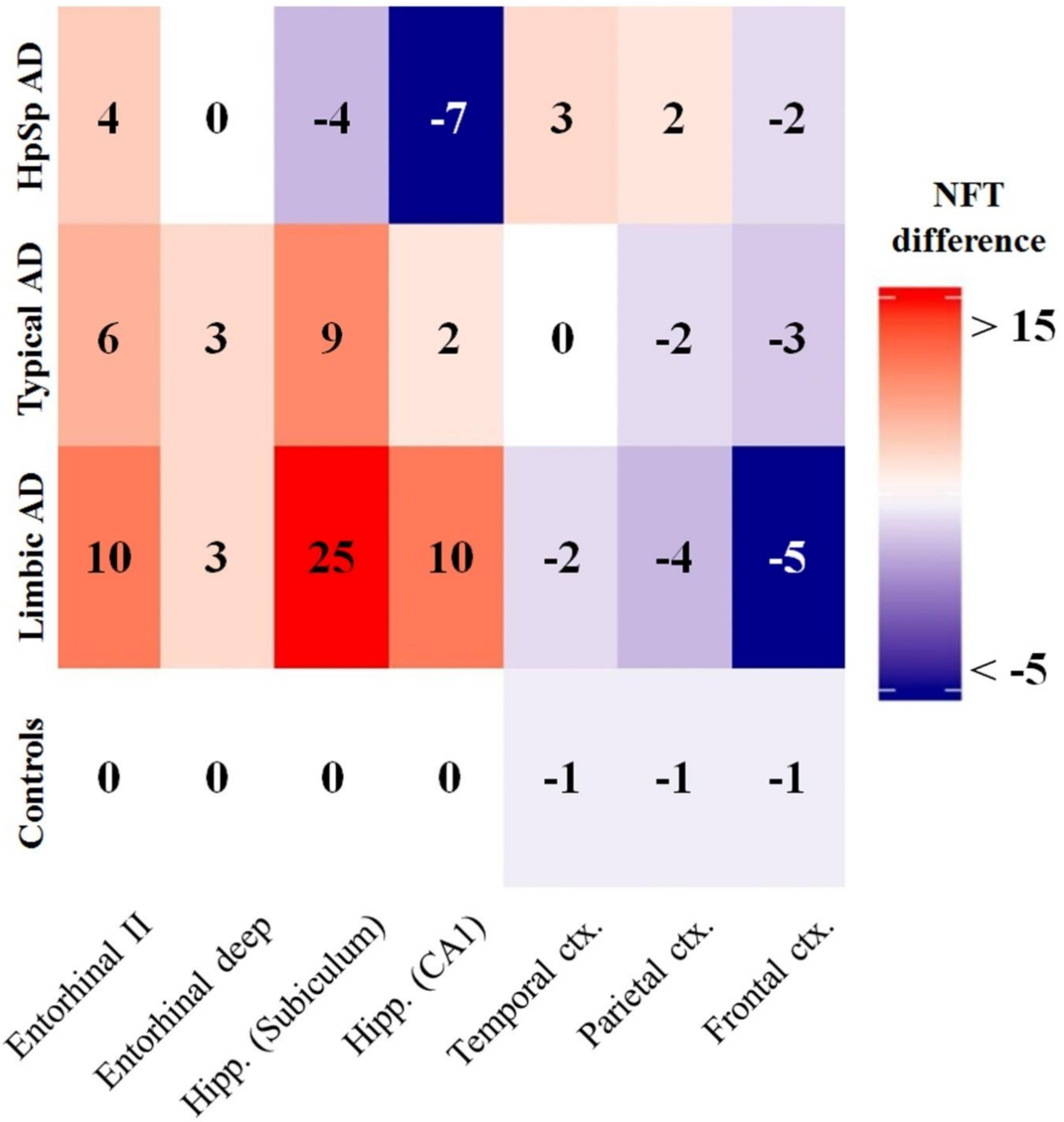

© 2019 Hanna Al-Shaikh FS et al. *JAMA Neurology*.

eTable 1. Sample Size Inclusion and Exclusion From Analyses Investigating Neurofibrillary Tangle Differences in the Nucleus Basalis of Meynert Within the Total Cohort of Normal Controls and Alzheimer's Disease Cases or by AD Subtype.

nbM NFT analyses	Total cohort (n=1464)		By AD subtype (n=1361)		
	Normal controls	All AD	HpSp AD	Typical AD	Limbic AD
**Total number of cases from Table 1**	**103**	**1361**	**175**	**1014**	**172**
**Cases included in nbM NFT analyses**	**88/103 (85%)**	**1263/1361 (93%)**	**163/175 (93%)**	**937/1014 (92%)**	**163/172 (95%)**
Anterior (AC decussation)	15	251	33	188	30
Anterior (AC split)	24	291	45	208	38
Anterointermediate	49	721	85	541	95
Cases excluded from nbM NFT analyses	***15/103 (15%)***	***98/1361 (7%)***	***12/175 (7%)***	***77/1014 (8%)***	***9/172 (5%)***
Tissue not available for study	*15*	*83*	*12*	*63*	*8*
Pre-Anterior	*0*	*5*	*0*	*5*	*0*
Intermediate	*0*	*10*	*0*	*9*	*1*

© 2019 Hanna Al-Shaikh FS et al. *JAMA Neurology*.

eTable 2. Sample Size Inclusion and Exclusion From Analyses Investigating Neuronal Differences in the Nucleus Basalis of Meynert Within the Total Cohort of Normal Controls and Alzheimer's Disease Cases or by AD Subtype.

nbM neuronal analyses	Total cohort (n=1464)		By AD subtype (n=1361)		
	Normal	All AD	HpSp AD	Typical AD	Limbic AD
**Total number of cases from Table 1**	**103**	**1361**	**175**	**1014**	**172**
**Cases included in nbM neuronal analyses**	**72/103 (70%)**	**1002/1361 (74%)**	**148/175 (85%)**	**727/1014 (72%)**	**127/172 (74%)**
Anterior (AC decussation)	12	176	27	132	17
Anterior (AC split)	23	244	42	171	31
Anterointermediate	37	582	79	424	79
Cases excluded from nbM neuronal analyses	***31/103 (30%)***	***359/1361 (26%)***	***27 /175 (15%)***	***287/1014 (28%)***	***45/172 (26%)***
Tissue not available for study	*31*	*352*	*27*	*280*	*45*
Pre-Anterior	*0*	*1*	*0*	*1*	*0*
Intermediate	*0*	*6*	*0*	*6*	*0*

© 2019 Hanna Al-Shaikh FS et al. *JAMA Neurology*.

eReferences

1. Mesulam MM, Geula C. Nucleus basalis (Ch4) and cortical cholinergic innervation in the human brain: observations based on the distribution of acetylcholinesterase and choline acetyltransferase. *J Comp Neurol.* 1988;275(2):216-240.
2. Mesulam MM. Cholinergic circuitry of the human nucleus basalis and its fate in Alzheimer's disease. *J Comp Neurol.* 2013;521(18):4124-4144.
3. Liu AK, Chang RC, Pearce RK, Gentleman SM. Nucleus basalis of Meynert revisited: anatomy, history and differential involvement in Alzheimer's and Parkinson's disease. *Acta Neuropathol.* 2015;129(4):527-540.
4. Mesulam MM, Mufson EJ, Levey AI, Wainer BH. Cholinergic innervation of cortex by the basal forebrain: cytochemistry and cortical connections of the septal area, diagonal band nuclei, nucleus basalis (substantia innominata), and hypothalamus in the rhesus monkey. *J Comp Neurol.* 1983;214(2):170-197.
5. Vogels OJ, Broere CA, ter Laak HJ, ten Donkelaar HJ, Nieuwenhuys R, Schulte BP. Cell loss and shrinkage in the nucleus basalis Meynert complex in Alzheimer's disease. *Neurobiol Aging.* 1990;11(1):3-13.
6. Murray ME, Graff-Radford NR, Ross OA, Petersen RC, Duara R, Dickson DW. Neuropathologically defined subtypes of Alzheimer's disease with distinct clinical characteristics: a retrospective study. *Lancet Neurol.* 2011;10(9):785-796.
7. Mesulam MM, Mufson EJ. Neural inputs into the nucleus basalis of the substantia innominata (Ch4) in the rhesus monkey. *Brain.* 1984;107 (Pt 1):253-274.
8. Braak H, Braak E. Neuropathological stageing of Alzheimer-related changes. *Acta Neuropathol.* 1991;82(4):239-259.

© 2019 Hanna Al-Shaikh FS et al. *JAMA Neurology*.
